# Association between PM_10_ and specific circulatory system diseases in China

**DOI:** 10.1038/s41598-021-91637-x

**Published:** 2021-06-09

**Authors:** Yifan Zhang, Yuxia Ma, Fengliu Feng, Bowen Cheng, Hang Wang, Jiahui Shen, Haoran Jiao

**Affiliations:** Key Laboratory of Semi-Arid Climate Change, College of Atmospheric Sciences, Ministry of Education, Lanzhou University, Lanzhou, 730000 China

**Keywords:** Environmental impact, Risk factors

## Abstract

Particulate matter (PM) has been proved to be a risk factor for the development of circulatory system diseases (CSDs) around the world. In this study, we collected daily air pollutants, emergency room (ER) visits for CSDs, and meteorological data from 2009 to 2012 in Beijing, China. After controlling for the long-term trend and eliminating the influence of confounding factors, the generalized additive model (GAM) was used to evaluate the short-term effects of PM_10_ on CSDs and cause-specific diseases. The results showed that for every 10 μg/m^3^ increase in PM_10_, the largest effect estimates in ER visits of total CSDs, arrhythmia, cerebrovascular diseases, high blood pressure, ischemic heart disease and other related diseases were 0.14% (95% CI: 0.06–0.23%), 0.37% (95% CI: − 0.23 to 0.97%), 0.20% (95% CI: 0.00–0.40%), 0.15% (95% CI: 0.02–0.27%), 0.18% (95% CI: 0.02–0.35%) and 0.35% (95% CI: − 0.04 to 0.79%), respectively. When NO_2_ or SO_2_ was added into the model, the effect estimates of PM_10_ were mostly attenuated, while in those models with PM_2.5_ added, the effect estimates of PM_10_ were mostly increased. Stratified analysis indicated that PM_10_ had a greater effect on males and the elderly.

## Introduction

Air pollution has been recognized as the world’s single-largest environmental health risk, which is closely related to the occurrence of diseases^1^. In the past few decades, the adverse effects of air pollutants have been widely illustrated in epidemiological studies worldwide^2,3^.

Particulate matter is a mixture of extremely small particles and droplets in the air, consisting of a variety of solid and liquid components such as organic and inorganic substances suspended in the air^4^. The presence of PM in the air leads to increased health risks^5–8^. In particular, particulate matter with aerodynamic diameters that are 10 µm and smaller (PM_10_) cannot be filtered out through the nose, cilia, or mucus of the respiratory tract^9^. Hence, they can reach the tracheobronchial and alveolar regions of the respiratory tract and eventually enter the circulatory system and cause diseases^10^.

Circulatory system diseases (CSDs) are the general term of cardiovascular and cerebrovascular diseases, which are the leading cause of death globally^11^. In recent years, a growing number of studies have reported that PM_10_ is associated with morbidity and mortality of CSDs. For example, a national study in the United States found that the multivariable-adjusted odds for the multiplicity of cardiovascular disease outcomes increased by 1.15 (95% CI: 1.07 to 1.22) times per 10 μg/m^3^ increase in PM_10_^5^. In Rome, Italy, Alessandrini et al. reported that per Inter-quartile range [IQR (19.8 μg/m^3^)] increase in PM_10_ concentration was associated with 2.64% (95% CI: 0.06 to 5.29) higher hospitalizations for cerebrovascular diseases^12^. In some Asian countries, such as Korea^13^, Thailand^14^ and Iran^15^, similar results have also been reported.

In China, studies conducted in big cities like Beijing^16^, Shanghai^17^, Guangzhou^18^, Hefei^19^, and Wuhan^20^ have examined the associations between PM_10_ concentrations and CSDs. However, most of the current studies are focused on the effects on broad categories of cardiovascular diseases. The associations between PM_10_ and ER visits for the cause-specific diseases are rarely reported. Given the different incubation periods and impact mechanisms of air pollution on different types of diseases, studying the general CSDs may underestimate the short-term effects of PM_10_ on certain types of diseases^21^.

To fill this gap, we explored the relationships between PM_10_ and ER visits for specific CSDs (including specifically, arrhythmia, cerebrovascular diseases, high blood pressure, ischemic heart disease and other related diseases) from 2009 to 2012 in Beijing. To our knowledge, few studies have looked at the relationship between PM_10_ and multiple specific diseases at the same time. Therefore, compared with previous studies, this study will give a more specific explanation of the adverse effects of PM_10_ and provide important outlook for public health research and management. In addition, since it is necessary to assess the adverse health effects of air pollution on potentially susceptible groups, sex and age stratified analyses are also considered in the study.

## Materials and methods

### Study area

Beijing (116º 25' E and 39º 54' N), the capital of the People’s Republic of China, is located in eastern China, with a total area of approximately 164,000 km^2^. Beijing has a sub-humid, warm temperate continental monsoon climate. The four seasons are distinct in Beijing, with a cold and dry winter and a hot and wet summer. In recent years, Beijing has undergone some serious air pollution due to rapid economic development and urban population expansion.

### Data collection

**Figure 1:**
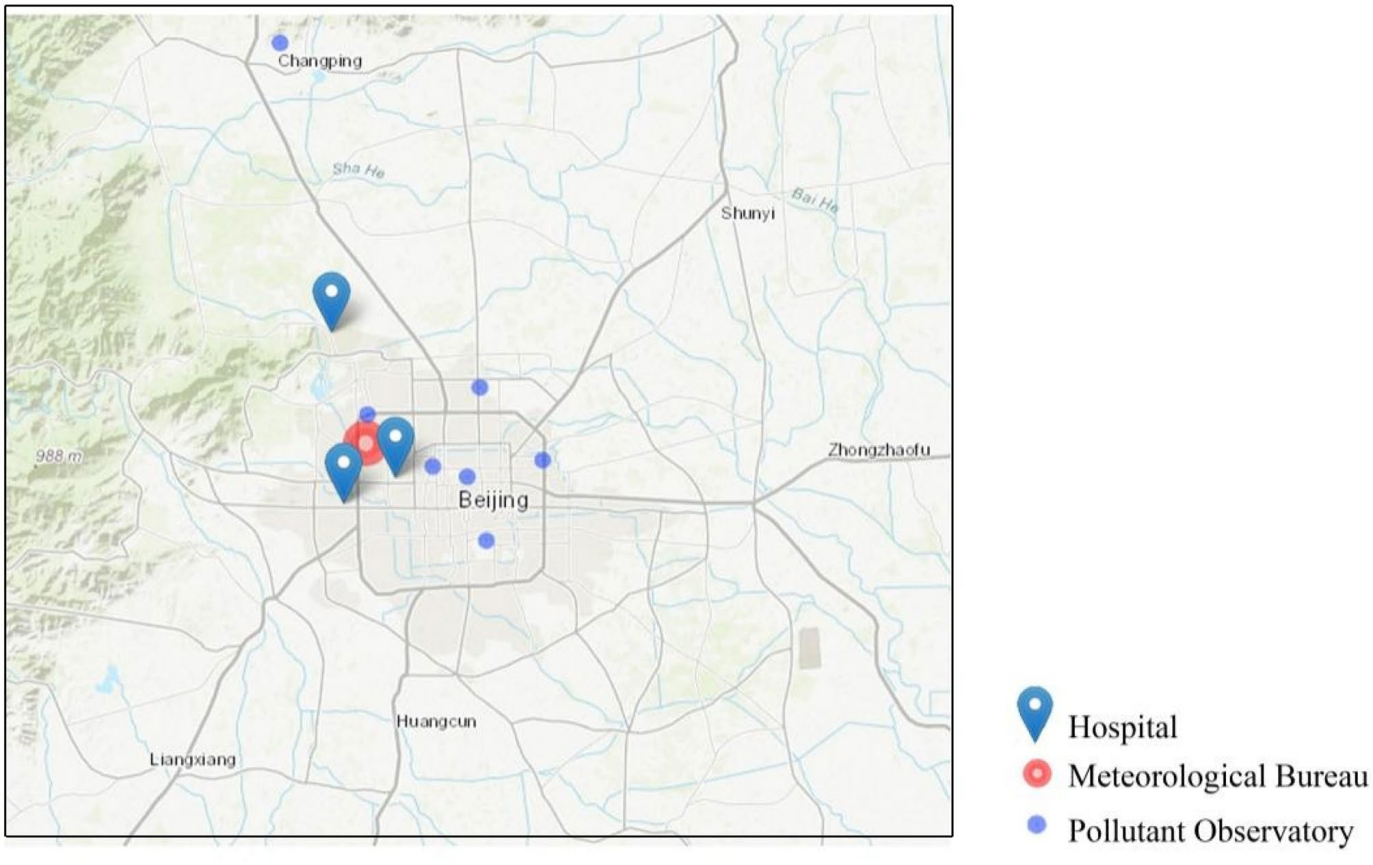
Locations of the three hospitals, the local Meteorological Bureau, and the seven pollutant observatories in the study area (R version 3.6.2 (2019-12-12) https://mirrors.tuna.tsinghua.edu.cn/CRAN/bin/windows/base/old/3.6.2/).

We obtained the daily records of ER visits for CSDs from January 1st, 2009, to December 31st, 2012, from three large-scale modern comprehensive hospitals in Beijing. They are General Hospital of the PLA (People's Liberation Army), the Sixth Medical Center of Chinese PLA General Hospital, and the Eighth Medical Center of Chinese PLA General Hospital. These three hospitals are all Third-level Grade A hospitals and cover about 10.82 million urban residents in Haidian, Xicheng, and Shijingshan districts (Fig. 1). Since these three hospitals have more professional diagnosis methods and treatment plans for CSDs, they are regarded as the first choice for local patients. The causes of daily ER visits from the three hospitals were all coded according to the tenth revision of the International Classification of Diseases (ICD-10). Specifically, visits associated with CSDs (I00–I99), arrhythmia (I44–I49), cerebrovascular disease (I60–I69), high blood pressure (I10), ischemic heart disease (I20–I25) and other related diseases were determined. Information such as age, sex and date of visit were also recorded. In the analysis, we divided the entire study group into two sex subgroups (males, females) and three age subgroups (aged 15–59 years, aged 60–74 years, and aged ≥ 75 years). We did not include the visit records of people under the age of 15 due to the relatively small sample size.

The daily average concentrations of air pollutants (PM_10_, PM_2.5_, NO_2_, SO_2_) were acquired from the average assessment of seven air quality monitoring stations in Beijing (Fig. 1). These stations, operated by the Ministry of Ecology and Environment of the People's Republic of China, were set up far from local pollution sources, and their results meet the Chinese government's quality assurance and control requirements and can reflect the general background of urban air pollution levels. We checked the quality of the data obtained, and the percentages of missing values for the four air pollutant concentrations were all less than 3%. The missing data were filled by interpolation in the analysis. Daily meteorological data (including average air temperature, air pressure, relative humidity, wind speed, and sunshine duration) during the study period were obtained from Beijing Meteorological Bureau. The average concentration of pollutants across the 7 monitoring stations and meteorological data from the Meteorological Bureau were aggregated to represent the daily exposure of the urban population. Everyday, patients visiting these three hospitals were assigned the same pollutant levels and meteorological data.

### Statistical methods

Because the number of daily hospital ER visits is a small probability event for the entire population of Beijing, it typically follows a Poisson distribution^22^. All data is public, there is no patient contact, and no PIN is required. Therefore, the study does not require ethical approval. In this study, we established a fitted Poisson distribution-based generalized additive model (GAM) (Eq. 1) to analyze the associations between the number of daily ER visits for CSDs and PM concentrations during the period of 2009–2012.

1 $$ {\text{Log}}\left[ {{\text{E(Y}}_{{\text{K}}} {)}} \right]{{ = \alpha +{\rm  DOW} + {\rm Holiday} + {\rm s} }}\left( {\text{time,df}} \right) +{\text{ s }}\left( {{\text{Z}}_{{\text{K}}} {\text{,df}}} \right){{ + \beta X}}_{{\text{K}}} $$

where $\text{E(}{\text{Y}}_{\text{K}}\text{)}$ refers to the expected ER visits on day *K*, ${\alpha}$ is the intercept, *DOW* denotes the day of week, *Holiday* is a created indicator function for Chinese holidays, *df* means the degree of freedom, *s (time, df)* refers to the spline function of calendar time, *s (*${\text{Z}}_{\text{K}}$*, df)* is the spline function of meteorological factors, ${\beta}$ is the regression coefficient, and ${\text{X}}_{\text{K}}$ indicates the concentrations of air pollutant on day *K*.

Establishing an appropriate GAM involved two steps: First, before including air pollutants, we used the spline function to remove the potential influence of confounding factors such as DOW, long-term trend, and meteorological factors. The Akaike’s Information Criterion (AIC) was applied to guide the selection of the smoothing df^23^. A smaller AIC value indicated a more suitable model. The detailed df values we used were given in the Appendix (Table S2). Second, we introduced air pollutants into the model to examine the lagging effects for 0–6 days (lag zero means the current day). Based on the estimated exposure–response coefficient $\beta$ in the GAM, we calculated the relative risk (RR) in the natural logarithm of the number of daily ER visits with per 10 µg/m^3^ increase in PM_10_ concentrations. We also calculated the “deviance explained” and “adjusted R^2^” to evaluate the predictive ability of the fitted model^24,25^. A larger value indicates that the model fits better (Appendix Table S1).

The robustness of the effect estimates was examined by using different lag structures [single-day lag (distributed lag: from lag 0 to lag 6); multiday lag (moving-average lag: lag 01 to lag 06)], testing the effects of PM_10_ on different subgroups [sex (females and males); age (15–59 years, 60–74 years and ≥ 75 years)], as well as estimating the effects of both single and multi-air pollutant models. In the multi-pollutant models, each pollutant was added to the single-pollutant model as a linear term at the optimal lag days. All the analyses were performed using the mgcv package in R 3.6.1 (R Foundation for Statistical Computing, Vienna, Austria).

## Results

From 2009 to 2012, a total of 79,259 CSDs ER visits were recorded in Beijing, including arrhythmia (2196), cerebrovascular disease (14,095), high blood pressure (35,601), ischemic heart disease (23,714), and other related diseases (3653). On average, the daily ER visits for CSDs and cause-specific diseases mentioned above were 54, 2, 10, 24, 16, and 3 respectively. The mean daily concentration of air pollutants was 130.06 μg/m^3^ for PM_10_, 70.71 μg/m^3^ for PM_2.5_, 57.12 μg/m^3^ for NO_2_, and 26.62 μg/m^3^ for SO_2_. PM_10_ was the major pollutant in Beijing, and its concentration on 73.5% of the days during the study period exceeded the National Grade II standard level (PM_10_: 70 μg/m^3^). Meanwhile, the daily average temperature, air pressure, relative humidity, wind speed, precipitation and sunshine duration were 13.08 °C, 1012.32 hPa, 50.54%, 2.23 m/s, 1.91 mm and 6.73 h respectively, reflecting the temperate continental monsoon climate of Beijing (Table 1).

**Table 1:** Descriptive statistics of air pollutants, meteorological factors, and emergency room visits in Beijing, China from 2009 to 2012.

Variable		$\overline{x }$ ± SD	Minimum	Maximum	Percentile		
					P_25_	P_50_	P_75_
Air pollutant concentrations	PM_10_ ($\upmu$g/m^3^)	130.06 $\pm$ 87.69	6.54	563.33	67.00	112.00	172.77
	PM_2.5_ ($\upmu$g/m^3^)	70.71 $\pm$ 56.77	3.00	381.55	28.29	58.00	98.02
	NO_2_ (μg/m^3^)	57.12 $\pm$ 25.65	11.20	241.60	38.40	52.54	71.29
	SO_2_ (μg/m^3^)	26.62 $\pm$ 27.47	0.19	234.50	8.00	17.00	34.73
Meteorological factors	Temperature ($^\circ$C)	13.08 $\pm$ 11.62	− 12.5	34.50	1.70	15.10	24.00
	Air pressure (hPa)	1012.32 $\pm$ 10.19	989.70	1037.30	1003.90	1011.80	1020.40
	Relative humidity (%)	50.54 $\pm$ 20.27	9.00	97.00	34.00	52.00	67.00
	Wind speed (m/s)	2.23 $\pm$ 0.92	0.60	6.40	1.60	2.10	2.70
	Precipitation (mm)	1.91 $\pm$ 0.20	0.00	82.90	0.00	0.00	0.00
	Sunshine duration (h)	6.73 $\pm$ 4.04	0.00	14.00	3.40	7.80	9.85
Emergency room visits	Arrhythmia	1.50 $\pm$ 1.48	0.00	10.00	0.00	1.00	2.00
	Cerebrovascular disease	9.65 $\pm$ 4.80	0.00	29.00	6.00	9.00	13.00
	High blood pressure	24.37 $\pm$ 7.91	2.00	57.00	19.00	24.00	30.00
	Ischemic heart disease	16.23 $\pm$ 6.66	1.00	44.00	11.00	16.00	21.00
	Other related diseases	2.50 $\pm$ 1.71	0.00	11.00	1.00	2.00	4.00
	Circulatory system diseases	54.25 $\pm$ 17.18	9.00	108.00	42.00	54.00	65.00

*SD* standard deviation.

**Figure 2:**
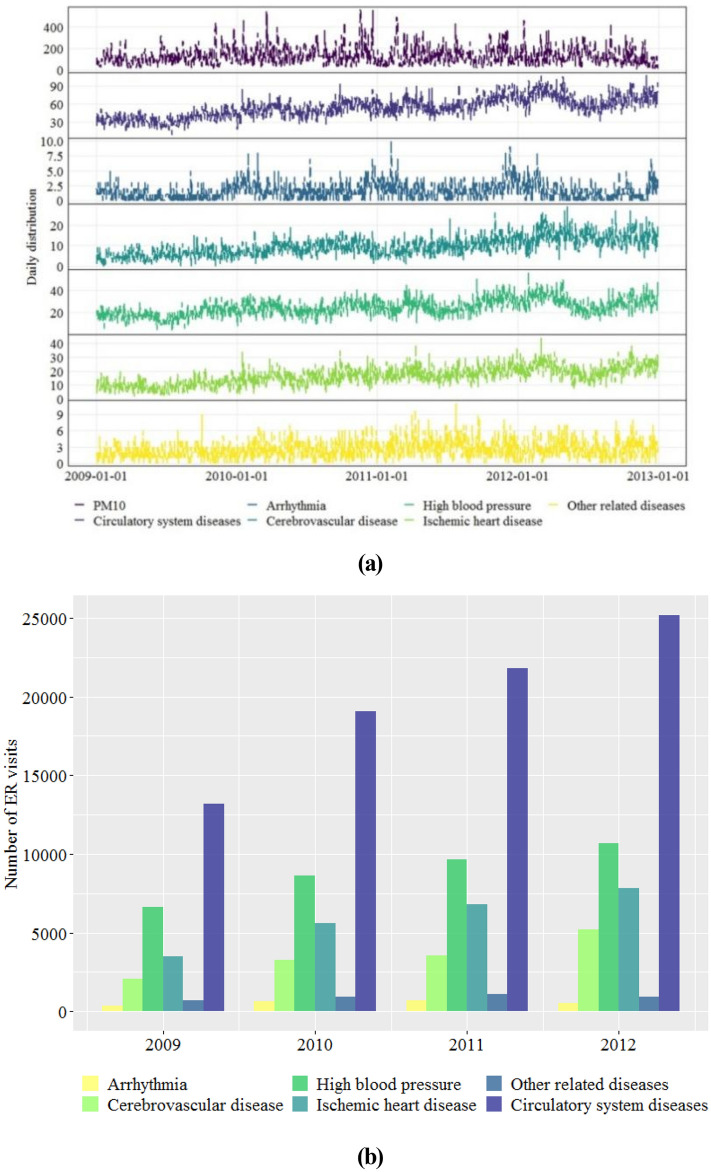
Time series of **(a)** PM_10_ and **(b)** emergency room visits for circulatory system diseases in Beijing, 2009–2012.

Figure 2 describes the time series of PM_10_ and CSDs ER visits during the study period. The concentration of PM_10_ increased in 2009–2010 and then declined slightly, but remained at a high concentration level. The number of ER visits for CSDs increased year by year and this trend was also found in visits due to cerebrovascular disease, high blood pressure, and ischemic heart disease. The number of ER visits for arrhythmia and other related diseases increased from 2009 to 2011, but declined slightly in 2012. Among the aforementioned diseases, high blood pressure was the top reason for ER visits (accounted for 44.92% of the total ER visits for CSDs), followed by ischemic heart disease (29.92%), cerebrovascular disease (17.78%), other related diseases (4.61%), and arrhythmia (2.77%).

The RRs of ER visits for each 10 μg/m^3^ increase in PM_10_ concentration varied by disease type, age, and sex. For the total population, the effects of PM_10_ on ER visits peaked at lag 2, lag2, lag3, lag1, lag0, lag1 for CSDs, high blood pressure, cerebrovascular disease, ischemic heart disease, arrhythmia, and other related diseases, with RRs of 1.0014 (95% CI: 1.0006–1.0023), 1.0015 (95% CI: 1.0002–1.0027), 1.0020 (95% CI: 1.0000–1.0040), 1.0018 (95% CI: 1.0002–1.0035), 1.0037 (95% CI: 0.9977–1.0097) and 1.0035 (95% CI: 0.9996–1.0079), respectively. The associations were all statistically significant except for ischemic heart disease and other related diseases. Strongest association was found between PM_10_ exposure and ER visits from arrhythmia at lag day 0, indicating that PM_10_ had a more acute pathogenic effect on the incidence of arrhythmia. The RRs of cerebrovascular disease visits were consistently above 1.000, suggesting that the influence of PM_10_ on this disease lasted for more than 6 days.

**Figure 3:**
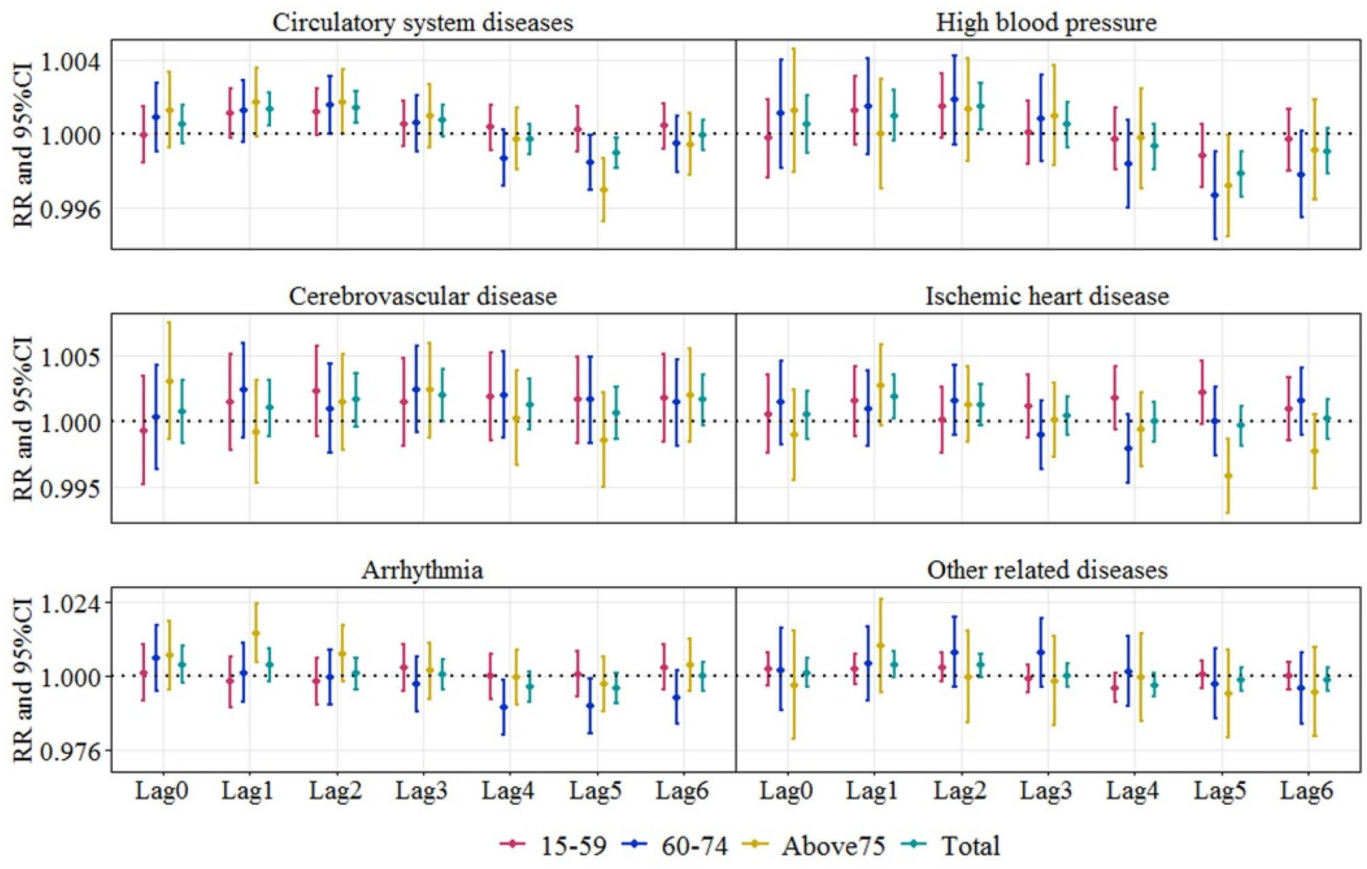
Relative risks s of emergency room visits for every 10 μg/m^3^ increase in PM_10_ under the age stratification.

**Figure 4:**
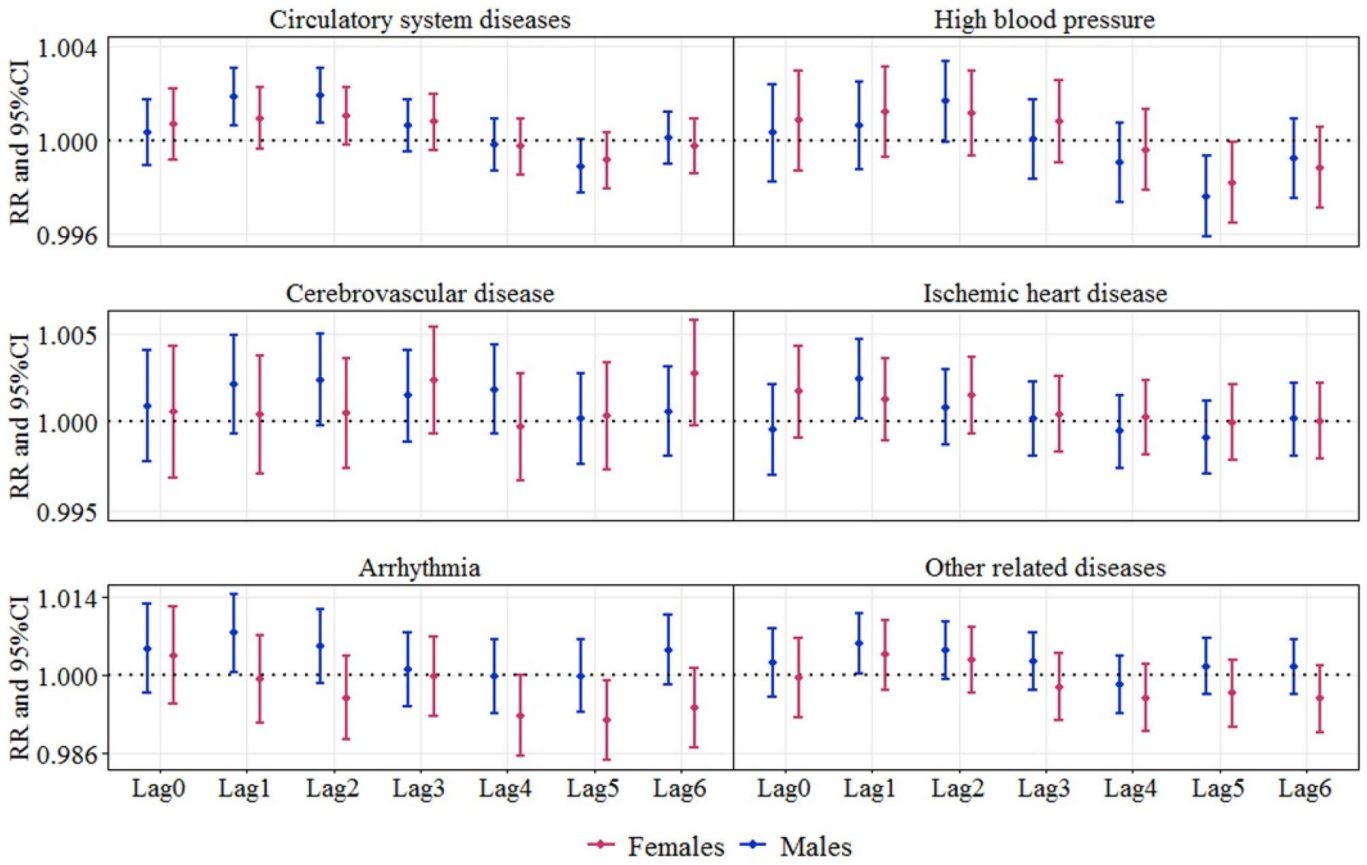
Relative risks (with 95% CI) of emergency room visits for every 10 μg/m^3^ increase in PM_10_ (lag 0–6) under the sex stratification.

Stratified analysis revealed the impacts of PM_10_ on different age and sex groups. Figure 3 shows the differences between age groups. For high blood pressure, higher estimated effects were found in people aged 60–74 years, while for the remaining diseases, the estimated effects were higher in people aged ≥ 75 years. This indicated that people aged 60–74 years were more affected by PM_10_ exposure in the incidence of high blood pressure, while people aged ≥ 75 years were more sensitive to the incidence of remaining diseases. Figure 4 illustrated the effect modification of PM_10_ risks by sex. In addition to cerebrovascular disease, PM_10_ was estimated to have greater impacts on CSDs and cause-specific diseases in males. These suggested males were generally more susceptible to aforementioned diseases than females, although the difference was not significant.

Table 2 shows the RRs of ER visits for per 10 μg/m^3^ increase in PM_10_ at multiday lags. The strongest effect of PM_10_ on ER visits for CSDs, high blood pressure, and ischemic heart disease were found at lag 03, and the RRs were 1.0021 (95% CI: 1.0008–1.0034), 1.0019 (95% CI: 0.9999–1.0038), and 1.0020 (95% CI: 0.9997–1.0043), respectively. For arrhythmia, cerebrovascular disease and other related diseases, the largest RRs were observed at lag 01, lag 06 and lag 02, respectively, with a RR of 1.0051 (95% CI: 0.9986–1.0117), 1.0042 (95% CI: 1.0005–1.0078) and 1.0051 (95% CI: 0.9997–1.0106).

**Table 2:** Relative risks of emergency room visits for every 10 μg/m^3^ increase in PM_10_ at multiday lags.

	Circulatory system diseases	Arrhythmia	Cerebrovascular disease
Lag01	1.0013(1.0002–1.0025)*	1.0051(0.9986–1.0117)	1.0013(0.9986–1.0040)
Lag02	1.0019(1.0007–1.0031)**	1.0045(0.9975–1.0115)	1.0019(0.9990–1.0047)
Lag03	1.0021(1.0008–1.0034)**	1.0041(0.9966–1.0116)	1.0030(0.9999–1.0061)
Lag04	1.0018(1.0004–1.0031)*	1.0017(0.9938–1.0097)	1.0035(1.0002–1.0067)*
Lag05	1.0011(0.9996–1.0025)	0.9996(0.9912–1.0080)	1.0035(1.0001–1.0070)*
Lag06	1.0009(0.9994–1.0025)	0.9993(0.9905–1.0081)	1.0042(1.0005–1.0078)*

	High blood pressure	Ischemic heart disease	Other related diseases
Lag01	1.0011(0.9994–1.0027)	1.0017(0.9996–1.0037)	1.0036(0.9986–1.0088)
Lag02	1.0018(1.0000–1.0036)*	1.0020(0.9999–1.0042)	1.0051(0.9997–1.0106)
Lag03	1.0019(0.9999–1.0038)	1.0020(0.9997–1.0043)	1.0046(0.9987–1.0105)
Lag04	1.0013(0.9992–1.0033)	1.0018(0.9993–1.0042)	1.0025(0.9962–1.0089)
Lag05	1.0000(0.9979–1.0022)	1.0015(0.9989–1.0040)	1.0020(0.9953–1.0088)
Lag06	0.9996(0.9973–1.0019)	1.0015(0.9988–1.0042)	1.0014(0.9943–1.0085)

**P < 0.01, *P < 0.05.

**Figure 5:**
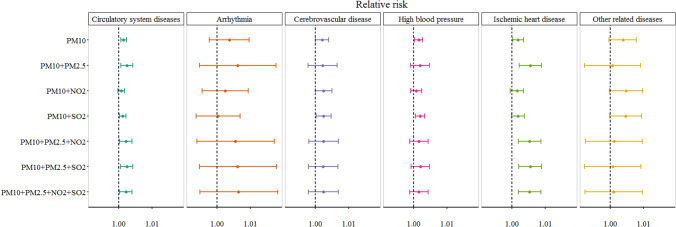
Effects of PM_10_ exposure on emergency room visits after adjusting for PM_2.5_, NO_2_, and SO_2_.

Besides, we examined the stability of the PM_10_ effects after adjusting for PM_2.5_, NO_2_, and SO_2_ (Fig. 5, Table S3). The results showed that compared with the single pollutant model, most of the effect estimates of PM_10_ were reduced when only gaseous pollutant NO_2_ or SO_2_ was added. In other cases, however, the estimated effects of PM_10_ have mostly increased.

**Figure 6:**
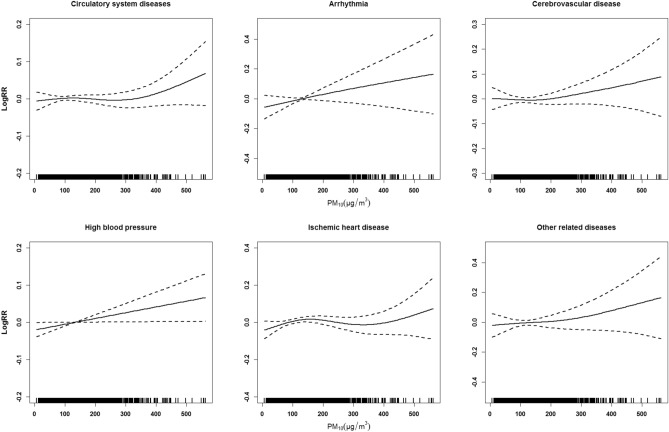
Exposure–response relationships of PM_10_ concentrations and emergency room visits for circulatory diseases and cause-specific diseases. The solid lines represent the logarithm of the number of emergency room visits, and the dotted lines indicate the 95% confidence intervals.

Figure 6 illustrates the exposure–response relationships between PM_10_ concentrations and ER visits. Generally, all the curves exhibited an upward trend. For arrhythmia and high blood pressure, the curves were approximately linear, indicating that a higher concentration of PM_10_ might cause a more significant increase in ER visits. The curves obtained for the cerebrovascular disease and other related diseases exhibited a similar trend. They showed a flat slope at low concentrations and then a slight increase as the concentration increased. The curves associated with CSDs and ischemic heart disease were slightly different from other curves. They tended to rise slightly at low PM_10_ concentrations, then flatten out, and continued to increase at higher concentrations. This nonlinear trend of sudden increase at the higher concentration was probably due to the data paucity at this range.

## Discussion

We investigated the impact of PM_10_ on CSDs and cause-specific diseases in Beijing from 2009 to 2012. The results showed that exposure to PM_10_ was associated with increased ER visits for CSDs, arrhythmia, high blood pressure, cerebrovascular disease, ischemic heart disease, and other related diseases. The effects were statistically significant at some lag structures. Among the aforementioned diseases, PM_10_ showed a more acute pathogenic effect on the incidence of arrhythmia. Stratified analysis indicated that the effects of PM_10_ appeared to be more evident in males and the elderly.

The association between PM_10_ and hospital visits has been well documented in developed countries, such as the USA^5,26^ and some European countries^27–29^. In recent years, studies in China have also described the adverse effects of PM_10_ on the incidence of circulatory diseases. In Shanghai, a study found that for every 10 µg/m^3^ increase in PM_10_ concentration, the risk of cardiovascular hospital admissions increased by 0.23% (− 0.03%, 0.48%)^17^. Another study provided the evidence that with the same increase in PM_10_, outpatient visits for arrhythmia increased by 0.56% (0.42%, 0.70%)^30^. In Guangzhou, Guo et al. investigated the short-term association between air pollutants and ER visits, indicating a 3.45% (1.09%, 5.86%) increase in circulatory diseases visits associated with PM_10_ exposure (per 45.51 µg/m^3^)^31^. In Beijing, a previous study we conducted showed that PM_10_ could lead to adverse cardiovascular outcomes. However, the study focused more on the effects of different types of pollutants such as PM_10_, SO_2_, and NO_2_ on the total CSDs rather than exploring the effects of these pollutants on specific types of diseases^32^. Feng et al. found positive associations between PM_10_ and emergency department admissions (EDAs) for cardiovascular diseases, including cerebrovascular events and ischemic heart disease^33^. For a 10 μg/m^3^ increase in PM_10_, the largest increase were 0.29% (0.12%, 0.46%), 0.36% (0.11%, 0.61%), 0.68% (0.25%, 1.10%) respectively. Guo et al. examined the relationship between particulate matter and the onset of hypertension, pointed that an increase in 10 μg/m^3^ in PM_10_ was associated with ER visits for hypertension with odds ratios of 1.060% (1.020, 1.101)^34^. The heterogeneity of effect estimates obtained in different studies could be explained by the differences in spatiotemporal changeability of air pollutant components and sources, sociodemographic variables, lifestyle of the studied populations, and factors used for controlling confounding biases^35–37^.

In this study, the strongest effects of PM_10_ were immediate or with a delay of up to 3 days. These results agree with other relevant studies that significant pathogenic effects of the pollutants were affected by the same-day pollution^38,39^ or pollution within a lag of 3 days^40^. Such the lag effects of particulate matter on human health could be explained by the fact that it takes time for a human body to accumulate pollutants to develop CSDs. We also found that the impacts of PM_10_ implicated different lag effects for different diseases. This may be due to PM_10_ triggers the onset of each disease in different ways. Also, different kinds of diseases may induce clinical manifestations with different severity, influencing the time lag of medical attendance^41^. Compared with other diseases studied, PM_10_ showed the most acute effect on the incidence of arrhythmia, which was coherent with a previous study that suggested an immediate effect of PM_10_ on arrhythmias^38^. We also found that PM_10_ had the greatest impact on arrhythmia visits. However, it is difficult to compare the results with previous studies because few studies have looked at the effects of PM_10_ on multiple specific diseases simultaneously.

Sex stratified analysis indicated that, except for cerebrovascular diseases, PM_10_ had a slightly higher effect on males than on females although this modification effect was not significantly different. This conclusion is consistent with other previous studies. For example, Phung et al. studied in Vietnam and found that when PM_10_ increased by 10 µg/m^3^, males had a higher risk of CVD admission (RR, 1.007, 1–1.01) than females (1.004, 1.001–1.007)^42^. Colais et al. conducted a study in 9 Italian cities suggested a stronger effect of ambient air pollution on arrhythmias and conduction disorders in males than in females^38^. Xu et al. in Shanghai, China observed a greater effect of PM_10_ on the risk of IHD in males compared to females^39^. Song et al. in Shijiazhuang also pointed out that particulate matter had stronger effects in men^43^. This may be due to the fact that outdoor workers are usually males, and compared with women, they usually have lower personal protective intentions (such as wearing masks)^44,45^. In addition, it has been reported that the habit of smoking also predispose men to the vulnerability of airway inflammation by PM_10_^46^. However, there are also some findings that differ from our conclusions, meaning that the sex-specific effects of PM_10_ remain controversial. For example, some studies have shown that associations between PM_10_ and admissions were not significantly modified by sex^40,47,48^, and some even pointed out that the effect of PM on human health was stronger in females than in males^30,49,50^. Such anecdotal conclusions could be explained by the possible fact that sex-specific effects might be caused by both socially derived gendered exposure and sex-linked biological differences (such as deposition of particles, airway responsiveness, and hormone statuses)^49,51^. Therefore, more evidence is needed to clarify this result. In terms of age, higher effect estimates were observed in people aged 60–74 years or ≥ 75 years, consistent with previous studies showed that the influence of PM_10_ on human health was more pronounced in the elderly^52,53^. In general, the elderly are considered to be more susceptible to air pollution because of their poor health condition and high prevalence of potential clinical conditions like preexisting heart problems^54^.

Effects of PM_10_ at the multiday lag models were analyzed to investigate the lag effect over time. In our study, the estimates using moving average lags were much higher than those using single-day lags. These results indicated that cumulative exposure to air pollutants increases the risk of morbidity, and using the single-day lag models alone might underestimate the cumulative association of PM_10_ with ER visits. Moreover, multi-pollutant models were used to examine the robustness of the results. After performing co-pollutant adjustment, almost all the estimated effects of PM_10_ were decreased after adjusting only for NO_2_ or SO_2_, while in other cases, most of the effect estimates were increased. This result suggested that the health effects of PM_10_ also affected by other pollutants, and due to the strong collinearity between pollutants, further studies are needed in the future to reveal the potential mechanism of pollutant interactions.

The mechanisms by which particulate matter affect the health of the circulatory system remains to be elucidated. Some studies have reported that ambient particulate matter can trigger cardiovascular events by affecting blood viscosity and vascular function (such as vascular dysfunction or vasoconstriction) directly^55,56^, or increase the cardiovascular burden indirectly by causing oxidative tension, inflammatory reactions and the release of activated leukocytes and cytokines in lung^57^.

The present study is one of the few studies that simultaneously investigated the effects of PM_10_ on CSDs and cause-specific diseases, which can help us understand the real incubation periods and effect estimates of certain diseases instead of overestimating or underestimating them. It also complements the aspects that were not covered in our previous study in this area, enabling us to more accurately assess the impact of PM_10_ on CSDs. However, there are also some limitations in our study. First, although we have controlled the long-term trend and eliminated the influences of confounding factors, we could not completely rule out the effects of individual differences such as exercise, diet, and other lifestyle factors. These factors will affect the individual's response to pollutants and lead to different effects of PM_10_. So our effect estimates may not be applicable to every individual in the city. Second, air pollutants data were obtained from fixed monitoring stations, which cannot reflect the true exposure of individuals. Since each person may have higher or lower exposure compared with the values from the monitoring stations, such exposure measurement error will cause the effect estimates to be overestimated or underestimated to vary degrees for each individual. Third, we only collected the hospital visits from three hospitals in Beijing, so the sample size may not be large enough to fully represent the entire population in the study area. Therefore, the results should be cautious when extrapolated.

## Conclusions

PM_10_ concentrations were positively associated with the number of ER visits for CSDs and cause-specific diseases in Beijing, China. Among the diseases studied, PM_10_ had a more acute pathogenic effect on the incidence of arrhythmia. Sex and age-stratified analysis showed that males and the elderly were more vulnerable to PM_10_ exposure. The results of this study could be used to assist local human health authorities in taking preventative measures in the long run.

## Supplementary Information

Supplementary Tables.
