# Supplementary material for: Association between PM10 and specific circulatory system diseases in China
Source: Sci Rep. 2021 Jun 9;11:12129. doi: 10.1038/s41598-021-91637-x (PMC8190074; doi:10.1038/s41598-021-91637-x)
Supplement: Supplementary file 1 — Supplementary Tables. [file 41598_2021_91637_MOESM1_ESM.docx]

Appendix

**Table S1.** **Evaluation of the established model's fitting for CSDs and cause-specific disease.**

|  | Deviance explained (%) | Adjusted R^2^ | AIC |
| --- | --- | --- | --- |
| CSDs | 68.4% | 0.666 | 11111 |
| Arrhythmia | 24.9% | 0.264 | 4430 |
| Cerebrovascular disease | 49.6% | 0.481 | 7647 |
| High blood pressure | 52.1% | 0.502 | 9233 |
| Ischemic heart disease | 56.2% | 0.542 | 8554 |
| Other related diseases | 11% | 0.097 | 5399 |

**Table S2. The degrees of freedom used in the model**

|  |  | Time | Temperature | Air pressure | RH | Wind speed | Precipitation | Sunshine duration |
| --- | --- | --- | --- | --- | --- | --- | --- | --- |
| CSDs | Total | 10 | 9 | 9 | 10 | 8 | 3 | 10 |
|  | 15-59 years | 9 | 9 | 4 | 3 | 6 | 3 | 5 |
|  | 60-74 years | 9 | 9 | 8 | 10 | 9 | 4 | 3 |
|  | ≥75 years | 10 | 6 | 9 | 9 | 4 | 3 | 7 |
|  | Male | 9 | 8 | 10 | 9 | 4 | 9 | 10 |
|  | Female | 10 | 10 | 9 | 8 | 7 | 10 | 7 |
| Arrhythmia | Total | 9 | 8 | 6 | 10 | 10 | 8 | 3 |
|  | 15-59 years | 7 | 4 | 4 | 4 | 3 | 9 | 3 |
|  | 60-74 years | 4 | 8 | 3 | 9 | 3 | 8 | 6 |
|  | ≥75 years | 10 | 8 | 5 | 9 | 10 | 4 | 3 |
|  | Male | 7 | 8 | 7 | 3 | 3 | 8 | 4 |
|  | Female | 8 | 8 | 3 | 5 | 6 | 7 | 3 |
| Cerebrovascular disease | Total | 9 | 4 | 10 | 9 | 10 | 10 | 10 |
|  | 15-59 years | 9 | 3 | 3 | 3 | 9 | 8 | 4 |
|  | 60-74 years | 9 | 10 | 10 | 3 | 10 | 9 | 10 |
|  | ≥75 years | 10 | 6 | 6 | 3 | 7 | 3 | 6 |
|  | Male | 9 | 10 | 3 | 9 | 3 | 5 | 3 |
|  | Female | 9 | 3 | 10 | 8 | 10 | 7 | 3 |
| High blood pressure | Total | 9 | 9 | 8 | 5 | 7 | 8 | 10 |
|  | 15-59 years | 8 | 9 | 4 | 8 | 10 | 5 | 3 |
|  | 60-74 years | 9 | 9 | 10 | 8 | 3 | 7 | 9 |
|  | ≥75 years | 10 | 8 | 5 | 3 | 6 | 4 | 3 |
|  | Male | 9 | 3 | 4 | 4 | 4 | 7 | 3 |
|  | Female | 9 | 10 | 5 | 8 | 4 | 4 | 10 |
| Ischemic heart disease | Total | 10 | 3 | 3 | 7 | 5 | 5 | 5 |
|  | 15-59 years | 7 | 4 | 3 | 4 | 9 | 6 | 6 |
|  | 60-74 years | 10 | 3 | 6 | 10 | 10 | 5 | 5 |
|  | ≥75 years | 10 | 4 | 5 | 3 | 4 | 9 | 6 |
|  | Male | 9 | 3 | 9 | 3 | 3 | 5 | 10 |
|  | Female | 10 | 3 | 3 | 4 | 3 | 5 | 5 |
| Other related diseases | Total | 10 | 7 | 6 | 10 | 10 | 8 | 10 |
|  | 15-59 years | 7 | 7 | 7 | 8 | 5 | 7 | 10 |
|  | 60-74 years | 7 | 4 | 3 | 7 | 7 | 3 | 3 |
|  | ≥75 years | 3 | 6 | 10 | 7 | 8 | 3 | 8 |
|  | Male | 10 | 7 | 3 | 10 | 10 | 9 | 10 |
|  | Female | 5 | 3 | 8 | 10 | 10 | 10 | 7 |

**Table S3. Effects of PM_10_ exposure on ER visits after adjusting for PM_2.5_, NO_2_, and SO_2_.**

|  |  | CSDs | Arrhythmia | Cerebrovascular disease |
| --- | --- | --- | --- | --- |
| Total | PM_10_ | 1.0014(1.0006-1.0023)** | 1.0037(0.9977-1.0097) | 1.0020(1.0000-1.0040)* |
|  | PM_10_ + PM_2.5_ | 1.0025(1.0006-1.0043)** | 1.0062(0.9947-1.0178) | 1.0022(0.9978-1.0066) |
|  | PM_10_ + NO_2_ | 1.0008(0.9997-1.0018) | 1.0024(0.9955-1.0094) | 1.0025(1.0000-1.0050)* |
|  | PM_10_ + SO_2_ | 1.0012(1.0002-1.0022)* | 1.0003(0.9937-1.0070) | 1.0025(1.0003-1.0048)* |
|  | PM_10_ + PM_2.5_ + NO_2_ | 1.0022(1.0003-1.0040)* | 1.0056(0.9940-1.0173) | 1.0024(0.9980-1.0069) |
|  | PM_10_ + PM_2.5_ + SO_2_ | 1.0024(1.0005-1.0043)* | 1.0062(0.9947-1.0179) | 1.0023(0.9979-1.0068) |
|  | PM_10_ + PM_2.5_+ NO_2_+ SO_2_ | 1.0022(1.0003-1.0040)* | 1.0065(0.9949-1.0183) | 1.0024(0.9979-1.0069) |
| Male | PM_10_ | 1.0019(1.0007-1.0031)** | 1.0075(1.0005-1.0145)* | 1.0024(0.9998-1.0050) |
|  | PM_10_ + PM_2.5_ | 1.0026(1.0001-1.0052)* | 1.0103(0.9952-1.0256) | 0.9991(0.9932-1.0051) |
|  | PM_10_ + NO_2_ | 1.0015(1.0000-1.0030)* | 1.0050(0.9961-1.0140) | 1.0020(0.9987-1.0054) |
|  | PM_10_ + SO_2_ | 1.0012(0.9999-1.0026) | 1.0041(0.9959-1.0124) | 1.0009(0.9979-1.0040) |
|  | PM_10_ + PM_2.5_ + NO_2_ | 1.0024(0.9998-1.0050) | 1.0093(0.9941-1.0247) | 0.9991(0.9931-1.0051) |
|  | PM_10_ + PM_2.5_ + SO_2_ | 1.0024(0.9999-1.0050) | 1.0102(0.9951-1.0255) | 0.9987(0.9928-1.0047) |
|  | PM_10_ + PM_2.5_+ NO_2_+ SO_2_ | 1.0024(0.9999-1.0050) | 1.0101(0.9949-1.0255) | 0.9991(0.9931-1.0051) |
| Female | PM_10_ | 1.0010(0.9998-1.0023) | 1.0035(0.9948-1.0122) | 1.0027(0.9998-1.0057) |
|  | PM_10_ + PM_2.5_ | 1.0030(1.0002-1.0057)* | 1.0115(0.9948-1.0285) | 0.9988(0.9919-1.0057) |
|  | PM_10_ + NO_2_ | 1.0002(0.9986-1.0018) | 1.0031(0.9930-1.0133) | 0.9966(0.9927-1.0005) |
|  | PM_10_ + SO_2_ | 1.0014(0.9999-1.0028) | 1.0000(0.9903-1.0098) | 1.0004(0.9968-1.0039) |
|  | PM_10_ + PM_2.5_ + NO_2_ | 1.0026(0.9998-1.0054) | 1.0112(0.9944-1.0283) | 0.9970(0.9900-1.0041) |
|  | PM_10_ + PM_2.5_ + SO_2_ | 1.0030(1.0003-1.0058)* | 1.0115(0.9947-1.0286) | 0.9988(0.9918-1.0057) |
|  | PM_10_ + PM_2.5_+ NO_2_+ SO_2_ | 1.0024(0.9996-1.0052) | 1.0121(0.9952-1.0293) | 0.9974(0.9904-1.0045) |
| 15-59 years | PM_10_ | 1.0011(0.9999-1.0024) | 1.0028(0.9955-1.0102) | 1.0023(0.9989-1.0057) |
|  | PM_10_ + PM_2.5_ | 1.0019(0.9991-1.0046) | 1.0067(0.9900-1.0237) | 1.0037(0.9961-1.0114) |
|  | PM_10_ + NO_2_ | 1.0010(0.9994-1.0026) | 1.0051(0.9952-1.0152) | 1.0031(0.9988-1.0074) |
|  | PM_10_ + SO_2_ | 1.0013(0.9999-1.0028) | 1.0020(0.9932-1.0109) | 1.0017(0.9978-1.0056) |
|  | PM_10_ + PM_2.5_ + NO_2_ | 1.0018(0.9990-1.0046) | 1.0070(0.9903-1.0240) | 1.0041(0.9964-1.0118) |
|  | PM_10_ + PM_2.5_ + SO_2_ | 1.0019(0.9992-1.0047) | 1.0067(0.9900-1.0237) | 1.0035(0.9959-1.0112) |
|  | PM_10_ + PM_2.5_+ NO_2_+ SO_2_ | 1.0018(0.9990-1.0046) | 1.0074(0.9907-1.0244) | 1.0041(0.9963-1.0118) |
| 60-74 years | PM_10_ | 1.0015(1.0000-1.0031) | 1.0057(0.9951-1.0165) | 1.0024(0.9991-1.0057) |
|  | PM_10_ + PM_2.5_ | 1.0025(0.9991-1.0060) | 1.0050(0.9839-1.0265) | 0.9987(0.9913-1.0061) |
|  | PM_10_ + NO_2_ | 1.0005(0.9985-1.0024) | 0.9982(0.9857-1.0108) | 1.0020(0.9978-1.0062) |
|  | PM_10_ + SO_2_ | 1.0006(0.9988-1.0024) | 0.9955(0.9839-1.0072) | 1.0030(0.9992-1.0067) |
|  | PM_10_ + PM_2.5_ + NO_2_ | 1.0021(0.9986-1.0055) | 1.0024(0.9809-1.0242) | 0.9984(0.9909-1.0059) |
|  | PM_10_ + PM_2.5_ + SO_2_ | 1.0023(0.9988-1.0057) | 1.0048(0.9837-1.0265) | 0.9988(0.9914-1.0062) |
|  | PM_10_ + PM_2.5_+ NO_2_+ SO_2_ | 1.0021(0.9986-1.0055) | 1.0032(0.9818-1.0251) | 0.9986(0.9911-1.0061) |
| ≥75 years | PM_10_ | 1.0017(1.0000-1.0034)* | 1.0140(1.0045-1.0236)** | 1.0030(0.9986-1.0074) |
|  | PM_10_ + PM_2.5_ | 1.0043(1.0005-1.0080)* | 1.0213(1.0015-1.0416)* | 1.0043(0.9962-1.0125) |
|  | PM_10_ + NO_2_ | 1.0007(0.9985-1.0029) | 1.0115(0.9999-1.0233)* | 1.0007(0.9957-1.0058) |
|  | PM_10_ + SO_2_ | 1.0016(0.9997-1.0036) | 1.0090(0.9981-1.0200) | 1.0020(0.9971-1.0069) |
|  | PM_10_ + PM_2.5_ + NO_2_ | 1.0038(1.0000-1.0076) | 1.0203(1.0003-1.0408)* | 1.0030(0.9947-1.0113) |
|  | PM_10_ + PM_2.5_ + SO_2_ | 1.0042(1.0005-1.0080)* | 1.0216(1.0015-1.0420)* | 1.0040(0.9958-1.0122) |
|  | PM_10_ + PM_2.5_+ NO_2_+ SO_2_ | 1.0038(1.0000-1.0076) | 1.0218(1.0016-1.0424)* | 1.0030(0.9947-1.0113) |

|  |  | High blood pressure | Ischemic heart disease | Other related diseases |
| --- | --- | --- | --- | --- |
| Total | PM_10_ | 1.0015(1.0002-1.0027)* | 1.0018(1.0002-1.0035)* | 1.0035(0.9996-1.0079) |
|  | PM_10_ + PM_2.5_ | 1.0019(0.9991-1.0047) | 1.0055(1.0022-1.0089)** | 1.0006(0.99218-1.0092) |
|  | PM_10_ + NO_2_ | 1.0007(0.9991-1.0024) | 1.0015(0.9995-1.0035) | 1.0048(0.9997-1.0098) |
|  | PM_10_ + SO_2_ | 1.0019(1.0005-1.0034)** | 1.0018(0.9999-1.0037) | 1.0046(0.9999-1.0094) |
|  | PM_10_ + PM_2.5_ + NO_2_ | 1.0016(0.9988-1.0044) | 1.0053(1.0019-1.0087)** | 1.0011(0.9925-1.0097) |
|  | PM_10_ + PM_2.5_ + SO_2_ | 1.0020(0.9992-1.0048) | 1.0055(1.0021-1.0089)** | 1.0007(0.9922-1.0093) |
|  | PM_10_ + PM_2.5_+ NO_2_+ SO_2_ | 1.0016(0.9988-1.0044) | 1.0053(1.0019-1.0087)** | 1.0010(0.9924-1.0096) |
| Male | PM_10_ | 1.0017(0.9999-1.0034) | 1.0024(1.0002-1.0047) | 1.0057(1.0003-1.0111)* |
|  | PM_10_ + PM_2.5_ | 1.0017(0.9979-1.0056) | 1.0055(1.0008-1.0102)* | 1.0027(0.9918-1.0137) |
|  | PM_10_ + NO_2_ | 1.0008(0.9986-1.0031) | 1.0016(0.9988-1.0044) | 1.0055(0.9990-1.0122) |
|  | PM_10_ + SO_2_ | 1.0018(0.9998-1.0038) | 1.0022(0.9996-1.0048) | 1.0057(1.0000-1.0118) |
|  | PM_10_ + PM_2.5_ + NO_2_ | 1.0014(0.9976-1.0053) | 1.0050(1.0002-1.0097)* | 1.0027(0.9917-1.0139) |
|  | PM_10_ + PM_2.5_ + SO_2_ | 1.0018(0.9980-1.0056) | 1.0054(1.0007-1.0101)* | 1.0027(0.9915-1.0141) |
|  | PM_10_ + PM_2.5_+ NO_2_+ SO_2_ | 1.0014(0.9975-1.0052) | 1.0050(1.0002-1.0098)* | 1.0027(0.9913-1.0141) |
| Female | PM_10_ | 1.0012(0.9993-1.0031) | 1.0017(0.9991-1.0043) | 1.0036(0.9974-1.0098) |
|  | PM_10_ + PM_2.5_ | 1.0026(0.9986-1.0065) | 1.0075(1.0026-1.0124)** | 1.0004(0.9875-1.0134) |
|  | PM_10_ + NO_2_ | 1.0024(1.0000-1.0048) | 1.0013(0.9983-1.0043) | 1.0063(0.9988-1.0140) |
|  | PM_10_ + SO_2_ | 1.0025(1.0003-1.0047)* | 1.0011(0.9982-1.0040) | 1.0040(0.9970-1.0112) |
|  | PM_10_ + PM_2.5_ + NO_2_ | 1.0031(0.9991-1.0071) | 1.0071(1.0022-1.0121)** | 1.0016(0.9888-1.0146) |
|  | PM_10_ + PM_2.5_ + SO_2_ | 1.0029(0.9989-1.0068) | 1.0074(1.0025-1.0124)** | 1.0004(0.9875-1.0136) |
|  | PM_10_ + PM_2.5_+ NO_2_+ SO_2_ | 1.0030(0.9990-1.0070) | 1.0073(1.0023-1.0122)** | 1.0016(0.9888-1.0146) |
| 15-59 years | PM_10_ | 1.0015(0.9998-1.0032) | 1.0022(0.9998-1.0045) | 1.0029(0.9984-1.0075) |
|  | PM_10_ + PM_2.5_ | 1.0011(0.9973-1.0049) | 1.0046(0.9993-1.0099) | 1.0027(0.9928-1.0127) |
|  | PM_10_ + NO_2_ | 1.0000(0.9978-1.0022) | 1.0029(0.9998-1.0059) | 1.0033(0.9975-1.0091) |
|  | PM_10_ + SO_2_ | 1.0019(0.9999-1.0039) | 1.0021(0.9994-1.0049) | 1.0011(0.9957-1.0065) |
|  | PM_10_ + PM_2.5_ + NO_2_ | 1.0006(0.9968-1.0044) | 1.0048(0.9994-1.0101) | 1.0028(0.9928-1.0128) |
|  | PM_10_ + PM_2.5_ + SO_2_ | 1.0012(0.9974-1.0050) | 1.0045(0.9992-1.0099) | 1.0024(0.9925-1.0125) |
|  | PM_10_ + PM_2.5_+ NO_2_+ SO_2_ | 1.0006(0.9968-1.0044) | 1.0048(0.9995-1.0102) | 1.0029(0.9930-1.0129) |
| 60-74 years | PM_10_ | 1.0018(0.9994-1.0042) | 1.0016(0.9989-1.0042) | 1.0078(0.9967-1.0189) |
|  | PM_10_ + PM_2.5_ | 1.0006(0.9953-1.0059) | 1.0092(1.0035-1.0150)** | 1.0048(0.9804-1.0298) |
|  | PM_10_ + NO_2_ | 1.0008(0.9977-1.0039) | 1.0011(0.9977-1.0045) | 1.0031(0.9890-1.0174) |
|  | PM_10_ + SO_2_ | 1.0013(0.9986-1.0041) | 1.0009(0.9978-1.0040) | 1.0018(0.9889-1.0148) |
|  | PM_10_ + PM_2.5_ + NO_2_ | 1.0003(0.9949-1.0057) | 1.0088(1.0030-1.0146)** | 1.0034(0.9787-1.0288) |
|  | PM_10_ + PM_2.5_ + SO_2_ | 1.0005(0.9952-1.0059) | 1.0090(1.0032-1.0148)** | 1.0033(0.9786-1.0287) |
|  | PM_10_ + PM_2.5_+ NO_2_+ SO_2_ | 1.0003(0.9949-1.0057) | 1.0088(1.0030-1.0147)** | 1.0031(0.9782-1.0286) |
| ≥75 years | PM_10_ | 1.0013(0.9985-1.0041) | 1.0027(0.9997-1.0058) | 1.0098(0.9949-1.0249) |
|  | PM_10_ + PM_2.5_ | 1.0040(0.9980-1.0101) | 1.0091(1.0029-1.0153)** | 1.0062(0.9744-1.0390) |
|  | PM_10_ + NO_2_ | 1.0014(0.9978-1.0049) | 1.0033(0.9996-1.0070) | 1.0118(0.9935-1.0305) |
|  | PM_10_ + SO_2_ | 1.0024(0.9992-1.0056) | 1.0034(1.0000-1.0069) | 1.0112(0.9944-1.0283) |
|  | PM_10_ + PM_2.5_ + NO_2_ | 1.0041(0.9980-1.0102) | 1.0091(1.0028-1.0153)** | 1.0043(0.9736-1.0361) |
|  | PM_10_ + PM_2.5_ + SO_2_ | 1.0043(0.9983-1.0103) | 1.0091(1.0029-1.0153)** | 1.0067(0.9749-1.0395) |
|  | PM_10_ + PM_2.5_+ NO_2_+ SO_2_ | 1.0040(0.9980-1.0101) | 1.0090(1.0028-1.0153)** | 1.0044(0.9736-1.0362) |
